# The Effect of Graphite Additives on Magnetization, Resistivity and Electrical Conductivity of Magnetorheological Plastomer

**DOI:** 10.3390/ma14237484

**Published:** 2021-12-06

**Authors:** Nursyafiqah Zaini, Norzilawati Mohamad, Saiful Amri Mazlan, Siti Aishah Abdul Aziz, Seung-Bok Choi, Norhiwani Mohd Hapipi, Nur Azmah Nordin, Nurhazimah Nazmi, Ubaidillah Ubaidillah

**Affiliations:** 1 Engineering Materials and Structures (eMast) iKohza, Malaysian-Japan International Institute of Technology, Universiti Teknologi Malaysia, Jalan Sultan Yahya Petra, Kuala Lumpur 54100, Malaysia; nursyafiqah96@graduate.utm.my (N.Z.); aishah118@gmail.com (S.A.A.A.); hiwani87@gmail.com (N.M.H.); nurazmah.nordin@utm.my (N.A.N.); nurhazimah@utm.my (N.N.); 2 Faculty of Engineering, Universiti Malaysia Sabah, Jalan UMS, Kota Kinabalu 88400, Malaysia; norzilawati@ums.edu.my; 3 Institute for Vehicle Systems and Engineering (IVeSE), Sultan Ibrahim Chancellery Building, Universiti Teknologi Malaysia, Jalan Iman, Johor Bahru 81310, Malaysia; 4 Department of Mechanical Engineering, The State University of New York, Korea (SUNY Korea), 119 Songdo Moonhwa-ro, Yeonsu-gu, Incheon 21985, Korea; 5 Department of Mechanical Engineering, Industrial University of Ho Chi Minh City (IUH), 12 Nguyen Van Bao Street, Go Vap District, Ho Chi Minh City 700000, Vietnam; 6 Mechanical Engineering Department, Universitas Sebelas Maret, J1. Ir. Sutami 36A, Kentigan, Surakarta 57126, Indonesia

**Keywords:** magnetorheological plastomer, graphite, magnetic property, resistivity, conductivity

## Abstract

Common sensors in many applications are in the form of rigid devices that can react according to external stimuli. However, a magnetorheological plastomer (MRP) can offer a new type of sensing capability, as it is flexible in shape, soft, and responsive to an external magnetic field. In this study, graphite (Gr) particles are introduced into an MRP as an additive, to investigate the advantages of its electrical properties in MRPs, such as conductivity, which is absolutely required in a potential sensor. As a first step to achieve this, MRP samples containing carbonyl iron particles (CIPs) and various amounts of of Gr, from 0 to 10 wt.%, are prepared, and their magnetic-field-dependent electrical properties are experimentally evaluated. After the morphological aspect of Gr–MRP is characterized using environmental scanning electron microscopy (ESEM), the magnetic properties of MRP and Gr–MRP are evaluated via a vibrating sample magnetometer (VSM). The resistivities of the Gr–MRP samples are then tested under various applied magnetic flux densities, showing that the resistivity of Gr–MRP decreases with increasing of Gr content up to 10 wt.%. In addition, the electrical conductivity is tested using a test rig, showing that the conductivity increases as the amount of Gr additive increases, up to 10 wt.%. The conductivity of 10 wt.% Gr–MRP is found to be highest, at 178.06% higher than the Gr–MRP with 6 wt.%, for a magnetic flux density of 400 mT. It is observed that with the addition of Gr, the conductivity properties are improved with increases in the magnetic flux density, which could contribute to the potential usefulness of these materials as sensing detection devices.

## 1. Introduction

In recent years, flexible electronic sensors have attracted considerable attention due to their multiple functions and promising applications. This kind of sensor is currently widely applied many practical applications such as in automotive [1], aerospace [2], manufacturing [3], and even human motion monitoring [4]. In contrast to conventional electronic sensors based on semiconductors and metallic materials, flexible strain sensors have the features of high sensing sensitivity [5], excellent stretchability [6], and the ability to detect both tiny and large motions [7]. Over the last two decades, a number of smart materials have attracted considerable attention in a wide range of engineering applications due to their unique and inherent properties. Electrorheological materials, electroactive polymers, and magnetorheological (MR) materials are examples of potential smart materials. MR materials belong to an important part of the field of smart materials; their rheological properties change rapidly in the presence of a magnetic field. More specifically, when the external magnetic field is applied, the rheological properties of MR materials can be quickly tuned or controlled, due to the formation of chain-like structures of particles in the field direction [8]. MR materials can be classified into different groups such as MR fluids (MRFs), MR elastomers (MREs), MR foams, MR greases (MRGs), MR gels, and MR plastomers (MRPs) [9,10,11,12,13,14]. 

Recently, MRPs have received growing attention due to their better stability and higher MR effect than other MR materials [15,16]. MRPs represent a new type of MR material and can be defined as a weakly cross-linked polymer consisting of micro-sized magnetic particles dispersed in a matrix. MRPs have the capability of changing their properties as a result of the application of an external stimulus, especially a magnetic field. Unlike conventional MR gel, MRP is a solid-like gel that functions like a plasticine. It can be made into different forms and the shapes can be maintained for a long time. In the early stages, MRPs normally consisting of non-magnetic responsive iron particles (3–5 μm) dispersed into a polymer matrix with low cross-linking, became promising candidates to substitute for traditional MR materials [17]. Carbonyl iron particles (CIPs) are widely used as a magnetic material due to the high saturation magnetization of the metallic elements, as well as high permeability and low residual magnetization. Due to the tunable electrical properties of MR materials, the study of their electrical properties has drawn the attention of a variety of scholars up to this point. Because of the presence of ferromagnetic particles (CIPs) in an insulating conductive matrix, MR materials can conduct electricity [16,17]. In the presence of a magnetic field, MR materials can transform from an electric insulator to an electric conductor [18,19,20]. Magnetic particles are dispersed randomly throughout the medium or matrix in the absence of the magnetic field and no conductive channels are formed, resulting in the material acting as an insulator [21]. Magnetic particles form a chain parallel to the applied magnetic field in the presence of the magnetic field. The stronger the applied magnetic field, the longer and tighter the chain that is formed. The material becomes an electrical conductor due to these complete chains, which create conductive paths for electron transfer [22,23,24]. However, the devising of excellent flexible electronic sensors utilizing MR materials requires complex design and costly materials [24].

It is known that the poor electrical conductivity, high resistivity, and low sensitivity of conventional electronic sensors have limited their use in diverse applications. Therefore, many attempts have been made to find viable candidate materials to overcome these limitations, by utilizing different types of matrix, e.g., polyurethane (PU), polyacrylamide, and polystyrene, and by the introduction of additives into the material, e.g., carbon nanotubes (CNTs) [25], carbon nanofibers (CFs) [26], and graphite (Gr) [27], to further improve the mechanical, chemical and electrical properties of MR materials. To date, PU has been widely used due to its better sedimentation stability [28]. However, PU has some drawbacks, such as poor compatibility with the hydrocarbon matrix, resulting in low electrical conductivity [29]. Recently, polyvinyl alcohol (PVA) has been introduced to overcome PU’s disadvantages and to achieve better mechanical performance, biocompatibility, and electrical conductivity, making it very useful in the development of sensor application systems [30]. As a result, PVA was used as a conductive matrix, and this is believed to improve the electrical conductivity [31]. In another study, PVA was used as a matrix with the addition of a Gr oxide additive, to examine the electrical conductivity. However, this research was focused on the rheological properties only. Hapipi et al. [32] utilized PVA as a matrix together with CIPs as magnetic particles, to investigate the rheological properties. The use of Gr in MR materials, particularly MREs, has been proven to enhance the electrical properties [19,33,34,35]. Bica [35] revealed that MRE is capable of becoming electroconductive with the addition of 14% Gr micro particles. This finding was supported by Huang et al. [36], who discovered that the Gr could change the function of MRE, making it either an insulator (<14%) or an electrical conductor (>14%). In terms of MRPs, Pang et al. [37] studied the rheological and conductive properties of a PU-based MRP with the addition of Gr. The results showed that the electrical conductivity of the MRP was increased by 10,000 times relative to pure MRP when 15 wt.% of Gr flakes were added. However, to the best of the authors’ knowledge, there is no report on the electrical characteristics of PVA-based MRPs. 

Consequently, the technical novelty and main contribution of this study is to investigate the electrical properties of Gr–MRPs, especially PVA-based MRPs. Although PVA may transmit electricity, its electrical conductivity is still too low for use as a potential sensor. As a result, carbon-based additions such as pure multiwalled carbon nanotubes, p-MWCNT, graphene, and Gr are most often utilized as additives to improve the conductivity of MRPs for possible applications in soft or flexible sensors. In this work, five different wt.% quantities of Gr additives were used to fabricate PVA-based MRPs, and the magnetization hysteresis loops of MRP and Gr–MRP were identified using a vibrating sample magnetometer (VSM). Subsequently, the two most significant electrical properties with regard to use in flexible sensors were investigated using five different Gr–MRP samples. It was demonstrated that the resistivity of the Gr–MRP decreased when the Gr content was increased up to 10 wt.% and the magnetic flux density was increased. However, it was shown that the conductivity of Gr–MRP increased when both the content of Gr additives and the magnetic flux density were increased. Therefore, the results investigated in this work can provide a useful guideline for devising high-performance flexible or soft sensors utilizing Gr–MRP smart material. 

## 2. Experimental

### 2.1. Raw Materials

Polyvinyl alcohol (PVA) was purchased from Merck Company, Darmstadt, Germany, with a molecular weight of 6000 ≥ 98.0% for use as a matrix. Dimethyl sulfoxide (DMSO) and sodium tetraborate decahydrate (borax) were obtained from a drug store; the latter for use as a cross-linking agent. CIPs (type OM) were selected as magnetic particles and obtained from Badische Anilin Soda-Fabrik (BASF) with size and density of 5 μm and 7.874 g/cm^3^, respectively. Gr powder with an average size of 16 μm and density of 1.8 g/cm^3^ was used as an additive and was purchased from R and M Chemicals, Evergreen Engineering and Resources Co., Semenyih, Malaysia. The aqueous solution was prepared using deionized water.

### 2.2. Fabrication of Samples

**Figure 1:**
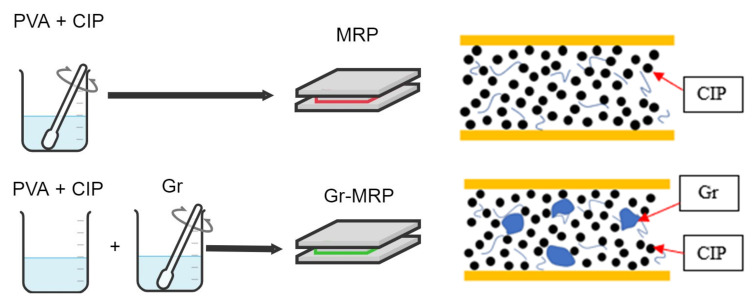
Fabrication process for MRP and Gr–MRP samples.

In order to fabricate the MRP samples, 70 wt.% CIPs and the PVA solution were mixed using a mechanical stirrer. However, in the case of the Gr–MRP samples, 70 wt.% CIPs and various amounts of Gr powder from 6 to 10 wt.% were mixed together with the PVA solution using a mechanical stirrer at 250 rpm until homogeneous, to produce samples 2 to 6. Table 1 presents the various compositions of the Gr–MRP samples. Both types of samples were prepared for a gelation process by stirring the mixture for another 10 min using a mechanical stirrer. In addition, a 3% (w/v) borax solution (cross-linking agent) was prepared by dissolving the borax powder in water. Then, the borax solution was added into the mixture and the mixture was then continuously stirred for another 10 min until gelation occurred. Lastly, the samples were kept overnight before testing to ensure the uniformity of the dispersion. The schematic diagram of the fabrication process of MRP and Gr–MRP is shown in Figure 1. 

### 2.3. Experimental Setup and Measurements

**Figure 2:**
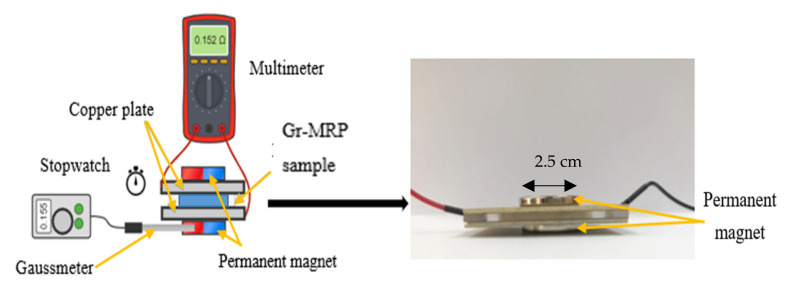
Experimental setup for measuring the resistance of the samples.

The resistivity was measured using an experimental setup that mainly consisted of a gaussmeter, a digital multimeter, copper plates, permanent magnets, and a stopwatch, as shown in Figure 2.

A sample was placed in the middle between the two copper plates attached to the permanent magnets. The copper plates acted as a pickup point for the electrical conductivity, while several permanent magnets were utilized to generate magnetic fields with various magnetic field strengths of 0, 0.1, 0.2, 0.3, and 0.4 T. After the connection was set up, the off-state condition (without applying a magnetic field) was measured using a fixed volume of the various samples. For the on-state condition (with a magnetic field), the test was carried out in the presence of permanent magnets with various magnetic field strengths. The resistance of the samples was measured using a Fluke 101 digital multimeter. Data representing the average of three resistance measurements are presented. In addition, a gaussmeter was utilized to determine the magnetic field strength at the samples.

### 2.4. Characterization Method

The surface morphology of the samples was examined using environmental scanning electron microscopy, ESEM (FEI, Quanta FEG, Holland, Netherlands), to investigate the distribution of the CIPs and Gr in the matrix. Platinum was used to coat the samples prior to the ESEM observation, in order to prevent electron charging of the sample surface resulting in glare on the images. The samples were analyzed at an accelerating voltage of 20 kV with magnifications of 1000×, 2500×, and 5000×. The magnetic properties of samples were measured using a vibrating sample magnetometer, VSM (Lake Shore 7404, Cryotronics, Westerville, OH, USA), within a continuous magnetic field range from −1100 to 1100 kA/m. All experiments were carried out at room temperature. The rheological properties of the MRP and Gr–MRP samples were measured under oscillatory mode using a rheometer (Physical MCR 302, Anton Paar, Graz, Austria) equipped with an external magneto-controllable accessory, MRD 70/1T. The sample was placed in a 20 mm diameter parallel plate with a 1 mm thickness gap and then placed in the cavity plate. For oscillatory mode, a shear strain was applied to the sample in the range of 0 to 100 Hz using a rotary disc parallel plate (pp20), and the strain was kept at 0.03%. The current was varied between 0 A, and 3 A during the test, which is equivalent to 0 and 540 mT, respectively.

## 3. Results and Discussion

### 3.1. ESEM Characterization

**Figure 3:**
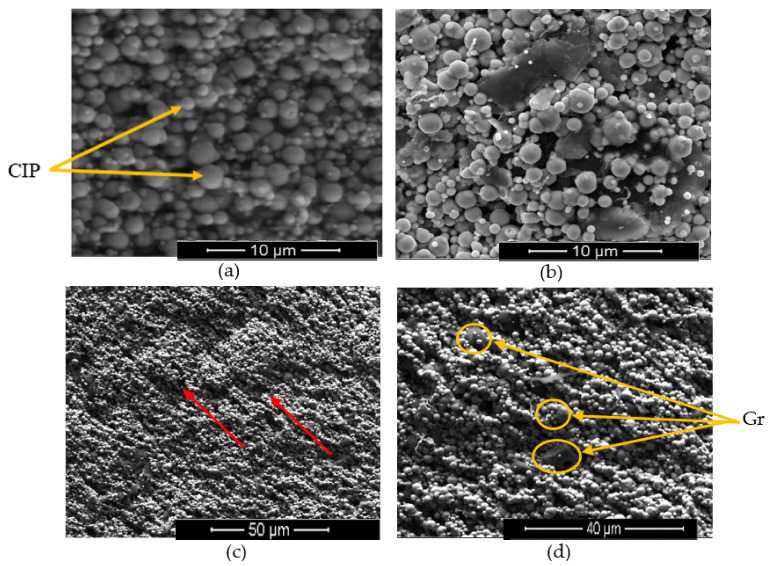
SEM images in the absence of magnetic field at magnification of 2500× for (**a**) MRP and (**b**) Gr–MRP. (**c**) Chain structure formation of Gr–MRP along with the presence of magnetic field at magnification of 500× and (**d**) enlargement of Gr in the presence of magnetic field. Red arrow represents the direction of magnetic field.

Figure 3 shows ESEM images for the distribution of CIPs in MRP and both CIPs and Gr in Gr–MRP, dispersed in a PVA matrix with and without the presence of a magnetic field. In this characterization investigation, only MRP (sample 1) and Gr–MRP sample 6 were selected for the comparison. 

As shown in Figure 3a, in the absence of a magnetic field, the CIP was randomly dispersed in the PVA matrix, while Figure 3b shows the dispersion of Gr and CIPs in the PVA matrix. It was observed that some of the CIPs were attached onto the surface of the Gr due to the large surface energy of Gr. This result agreed well with the findings of Zhang et al. [33]. However, Figure 3c depicts the CIPs and Gr distributions in the presence of the magnetic field. The CIPs and Gr tended to form chain-like structures in the PVA matrix with low cross-linking, parallel to the direction of the magnetic field. Although the magnetic field had no particular effect on the non-magnetic Gr, the CIPs attached to the Gr showed a tendency to align with other CIPs due to the magnetic force. Consequently, chain structures were constructed by the combination of both CIPs and Gr. In contrast to the case in MREs, the CIPs in the MRP are not permanently attached in the polymer matrix; therefore, they cannot closely attach to one another, resulting in many gaps [37].

**Figure 4:**
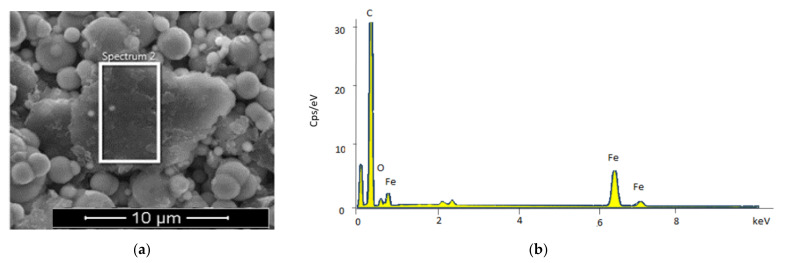
ESEM image of Gr–MRP sample with selected microstructure area (**a**) and the EDX observation of the Gr–MRP sample (**b**).

Figure 4a shows a selected area of the ESEM micrograph of Gr–MRP, confirming the elemental composition through EDX analysis. Three types of elements: carbon (C), oxygen (O), and iron (Fe) were detected in amounts of 73.56, 7.18, and 19.26 wt.%, respectively. Obviously, the carbon atom structure from CIPs and Gr contributed the highest proportion of C. According to Sengupta et al. [27], the Gr structure consists basically of two-dimensional (2D) lattice bonds, which is the most stable kind of carbon under standard conditions. The Fe element was obtained from the dominant composition of CIPs, while the O element occurred due to the formation of oxide during the thermal decomposition process at the manufacturer [34].

### 3.2. Magnetic Properties

**Figure 5:**
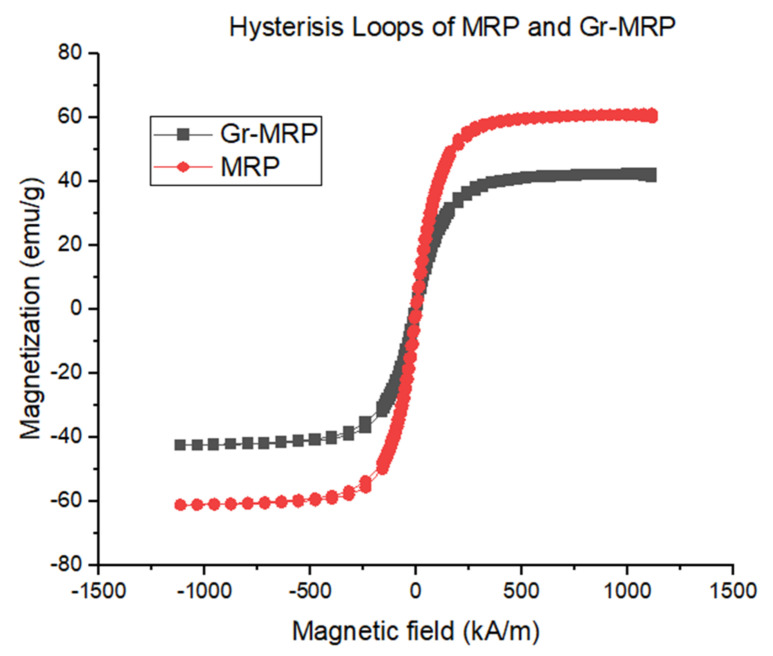
Magnetization curves of MRP and Gr–MRP samples.

Figure 5 shows the narrow hysteresis loops of typical soft magnetic material behavior for both the MRP and Gr–MRP samples. The magnetization properties were measured using a vibrating sample magnetometer (VSM) over a wide range of magnetic fields from −1100 to 1100 k/Am at room temperature. 

Magnetic properties such as coercivity (H_c_), retentivity (M_r_), and magnetic saturation (M_s_) are shown in Table 2. The coercivity values (H_c_) for both MRP and Gr–MRP were the same, at 7.97 kA/m. H_c_ is defined as the value of the magnetic field required to bring the magnetization back to zero. A small value of H_c_ indicates good soft magnetic properties, which are desirable in the fabrication of smart materials as the magnet is easily demagnetized after removing the magnetic field. It should be noted that sample 6 could represent all the Gr–MRP samples (samples 2, 3, 4, and 5) since the content of CIP was the same at 70% and a few studies [38,39] have shown that the Gr does not substantially influence the magnetic properties, especially M_s_. The magnetization saturation was therefore mostly determined by the CIP content of MRP and Gr–MRP samples. 

The M_s_ for the MRP sample was higher than for the Gr–MRP sample, at 61.15 emu/g compared to 42.45 emu/g. A higher value of M_s_ for MRP demonstrated a better ability of the material to respond to the applied magnetic field. In other words, a higher magnetic field strength, attributed to stronger interparticle interactions within CIPs, led to better alignment of the CIPs. In the Gr–MRP sample, the M_s_ dropped by 45% in comparison to the MRP sample, due to the Gr particles, which might have hindered and obstructed the movement of CIPs. Thus, the interparticle interactions were reduced, resulting in a smaller value of M_s_. In addition, due to the higher aspect ratio caused by the increased average diameter of the Gr, the Gr interfered with the accompanying CIPs formation of a chain-like structure on excitation by the magnetic field. Hence, since the Gr is not a magnetic material, the existence of Gr between the CIPs distorted the domain dipole interaction, thus reducing the M_s_. The same trend was observed for the M_r_ values, which were 200.67 × 10^−2^ and 116.58 × 10^−2^ emu/g for the MRP and Gr–MRP samples, respectively. In general, the M_r_ value indicates the value of the magnetic moment of the material that is lost after removing the magnetic field. The results revealed that the magnetism of particles did not remain after the magnetic field was removed. Therefore, a lower value of M_r_ in Gr–MRP was due to less interaction between CIPs in the sample owing to the presence of Gr, making it easier for the particles to self-demagnetize.

### 3.3. Resistivity Properties

**Figure 6:**
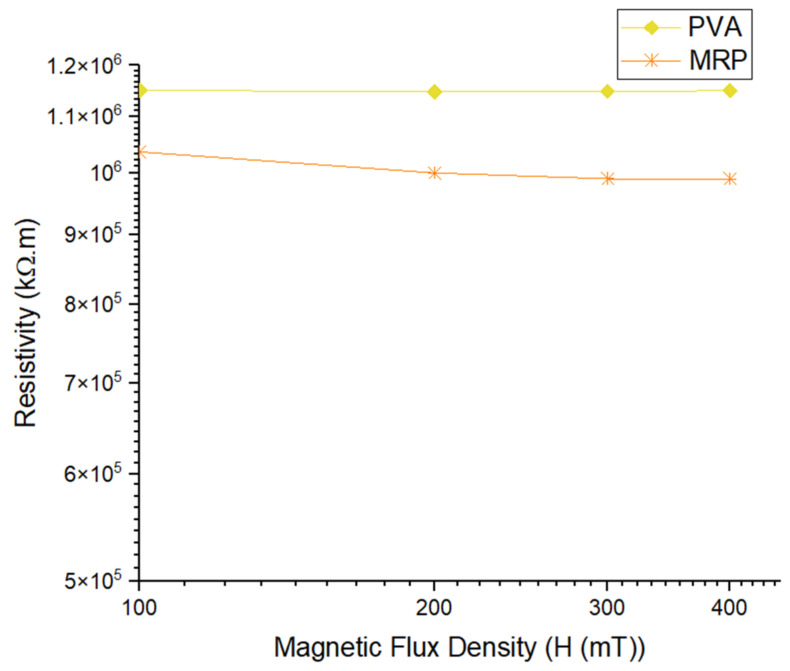
Resistivity of PVA and MRP under different magnetic flux densities.

The resistivity values with increasing applied magnetic field for both PVA and MRP samples are shown in Figure 6. The resistivity values for PVA were almost unchanged, while for MRP they decreased on increasing the magnetic flux density from 0 to 400 mT.

Figure 6 shows the resistivity trend of PVA and MRP under different magnetic flux densities. The resistivity of MRP was found to be lower than that of PVA and decreased as the magnetic flux density increased, whereas PVA’s resistivity remained constant over the magnetic flux density range. In other words, in the absence of magnetic flux density (0 mT), the initial resistivity of the PVA sample was considerably higher than that of the MRP sample and it had a constant value over the magnetic flux density range. For MRP, the initial resistivity value was lower than for the PVA sample, and in the on-state condition, the resistivity values decreased as the magnetic flux density increased. The resistivity trend for the MRP sample reached a plateau at higher magnetic flux densities, known as saturated resistivity. The results showed that the external magnetic flux density had no significant influence on the PVA sample but seemed to have a significant effect on the resistivity of the MRP sample. The resistivity values for both samples are shown in Table 3.

The initial resistivity of PVA at 0 mT was 1.15 × 10^6^ kΩ·m and the value remained constant at a higher magnetic flux density of 400 mT. The initial resistivity of MRP was 1.15 × 10^6^ kΩ·m, which was lower than that of PVA, and the value continued to decrease until it reached 9.90 × 10^5^ kΩ·m at 400 mT. The smaller value of the resistivity for MRP samples occurred due to the existence of CIPs as magnetic particles that simultaneously influenced the resistivity of the sample under the influence of the magnetic flux density. Despite the fact that CIP is considered a magnetic material, it has a resistance value that results in a contribution to the electrical conduction [40]. The resistivity values of MRP decreased slightly with a further increase in magnetic flux density, and eventually reached saturation at 300 mT. Although PVA is a conducting matrix, PVA had a consistent trend since it was not affected by the magnetic field. On the other hand, the small decreasing trend in MRP occurred because of the magnetic field, which caused the magnetic particles to instantaneously exhibit a stable dipole–dipole interaction between the particle structures. Furthermore, the changes in the conductive network owing to the PVA matrix might be one of the factors contributing to the decreasing trend in resistivity. 

**Figure 7:**
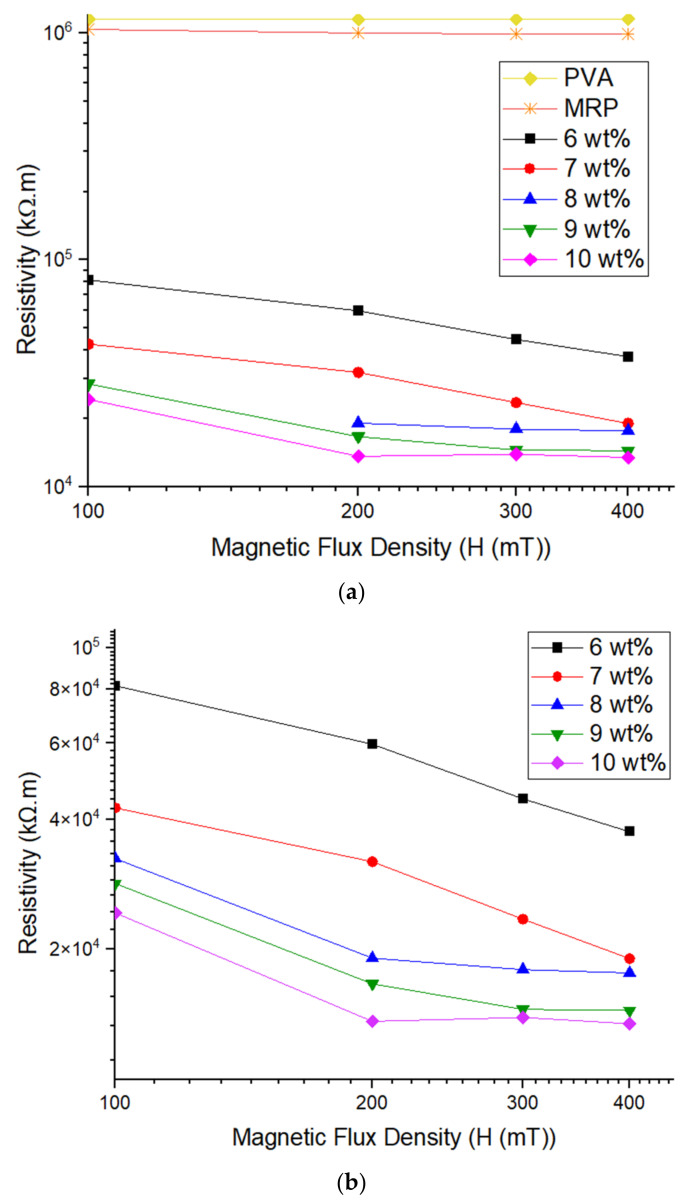
Resistivity trends of PVA, MRP, and five different weight percentages of Gr–MRP. (**a**) Resistivity of all samples and (**b**) enhanced resistivity scale for samples with different Gr contents under different magnetic flux densities.

In order to investigate the effect of Gr on the resistivity of Gr–MRP, samples with different Gr content (samples 2 to 6) were evaluated, as shown in Figure 7. Figure 7a shows the resistivity of all samples, i.e., PVA, MRP (sample 1), and Gr–MRP (samples 2–6) under various magnetic flux densities from 0 to 400 mT. All samples exhibited decreasing resistivity trends except for PVA, where the magnetic flux density had no effect on the resistivity as it was increased.

Further evaluation of the effect of Gr on resistivity is shown in Figure 7b by focusing on samples 2 to 6. The initial resistivity gap between Gr–MRP with PVA and MRP samples was large, due to the existence of the Gr in the MRP, which has a high number of free delocalized electrons that can move freely, leading to high conductivity. It was noted that the resistivity of the Gr–MRP samples decreased with increasing magnetic flux density. The trend was for a rapid decrease at lower magnetic flux densities until a plateau was reached at higher magnetic flux densities. At 200 mT, the resistivity values of samples 4 and 5 became constant. The point of saturation resistivity for Gr–MRP varied slightly depending on the weight percentage of Gr. The resistivity for a lower Gr content saturated at a low magnetic field density, while for a higher Gr content it saturated at a high magnetic flux density. A summary of the resistivity values of various weight percentages of Gr is shown in Table 4.

In general, the resistivity of Gr–MRP samples in the off-state condition (0 mT) was higher in comparison to the values in the on-state conditions (>0 mT). The higher the Gr content, the lower the observed resistivity values. However, remarkable changes in the initial resistivity value were observed for Gr–MRP samples higher than 7 wt.%. This might be due to a higher content of Gr in the MRP, thus resulting in an enhancement of the continuity of the conducting network. Moreover, in the absence of the magnetic flux density, the CIPs inside the Gr–MRP samples were randomly dispersed, thus resulting in agglomeration of the CIPs and Gr particles. However, in the on-state conditions (>0 mT), the resistivity value decreased with an increase in both Gr content and the applied magnetic field. This phenomenon occurred due to the strong magnetic force on the chain-like structures formed, which resulted in a reduction in the resistivity in Gr–MRP samples.

**Figure 8:**
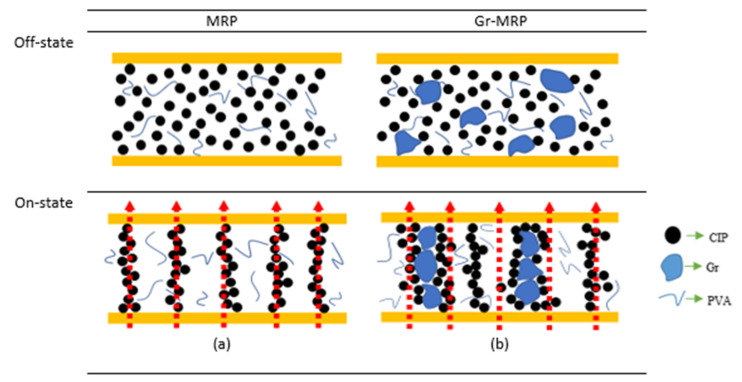
Illustration of magnetoresistance phenomena during the off-state (absence of magnetic field) and on-state (presence of magnetic field) conditions for (**a**) MRP and (**b**) Gr–MRP samples. Red arrow represents the direction of magnetic field.

Figure 8 illustrates the particles inside the MRP and Gr–MRP samples in the off- and on-state conditions. As depicted in Figure 8a, the CIPs are dispersed randomly in the PVA matrix in the off-state condition in the MRP sample. In the on-state conditions, the CIPs tended to align and form a “new network” with a chain-like structure, according to the magnetic field direction [32]. Since the CIPs are a weakly conducting material, low resistivity values of MRP in the off-state condition were obtained. For the Gr–MRP samples in the off-state condition, the CIPs and Gr were assumed to be dispersed randomly in the matrix, as shown in Figure 8b. However, the presence of Gr, which acts as a conducting material, led to a decrease in resistivity, enhancing the conductivity compared to the MRP samples. In the on-state conditions, the CIPs tended to align along the magnetic field direction, as in the MRP. However, since some of CIPs were attached to Gr, they also had a tendency to align, along with the other CIPs, creating a new combined structure as shown in Figure 3b,c. In fact, due to the rough and large-surface-area contact for irregular Gr, more CIPs accumulated around the Gr surface. The new structure formation phenomenon could be also related to the movement of CIPs aligning along the magnetic field direction, where the Gr has filled the space during the movement. As a result, the Gr moves and creates a more structured distribution, thus allowing more current to pass through the structure. This phenomenon was also observed by Nasir et al. [41].

In other words, the CIPs were used in the alignment process, and the Gr followed the movement of the CIPs that were attached to it. As a result, the Gr tended to “move” with the magnetizable CIPs, becoming involved in the formation of a columnar chain structure of the CIPs and Gr within the matrix, resulting in improved and stronger contact between them. This interfacial interaction between the matrix and the additives resulted in a conductivity enhancement, as reported by Shabdin et al. [42]. This reduction in resistivity as the magnetic flux density increased might be due to particle mobility influencing the CIP content and therefore the movement of Gr particles under various magnetic flux strengths. Moreover, the effective interfacial interaction between the filler and matrix, where a stronger interaction between the particles has increased the particle magnetization, resulted in an increment in the conductivity and a reduction in the resistance. As is widely known, the relationship between resistivity (σ) and electrical conductivity (ρ) is inversely proportional, as given by Equation (1),

(1) $$\sigma =\frac{1}{\rho }$$

**Figure 9:**
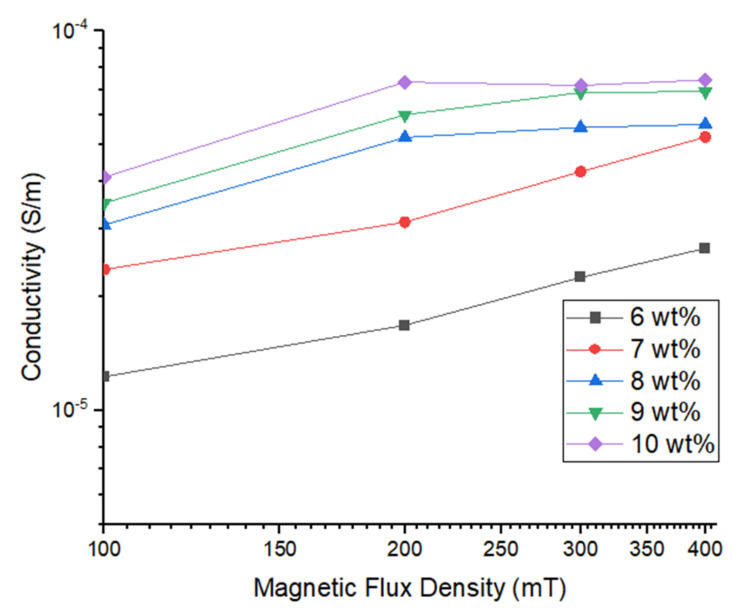
Conductivity of all samples with various Gr contents under different magnetic flux densities.

Thus, low resistivity implies a material that permits electric current to flow freely. As the resistivity increases, the electrical conductivity decreases. Figure 9 shows the conductivity of the samples.

Figure 9 shows the conductivity trends, where the resistivity values are fitted into the equation against various magnetic flux density values from 0 to 400 mT. The conductivity of 10 wt.% Gr–MRP was found to be the highest, at 178.06% higher than the Gr–MRP with 6 wt% at a magnetic flux density of 400 mT. In the absence of the magnetic flux density (0 mT), the initial conductivity values of all Gr–MRP samples were slightly increased with an increase in Gr content in the MRP. In general, as the magnetic flux density increases, the conductivities of all Gr–MRP samples tended to increase also. However, for higher Gr contents (8, 9 and 10%) at magnetic flux densities greater than 200 mT, the rate of increase in conductivity started to decline until a saturation phase was reached. Thus, it was evident that the addition of Gr to the samples could enhance the electrical conductivity.

### 3.4. The Effect of Frequency on MRP and Gr–MRP

**Figure 10:**
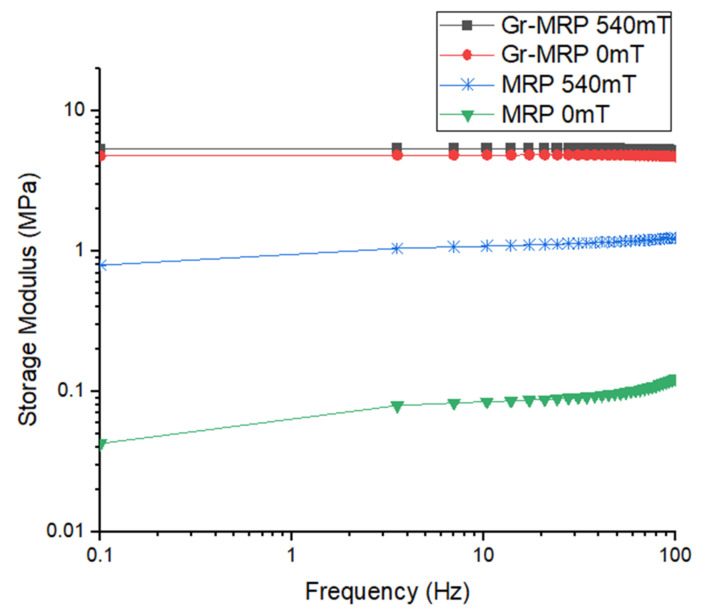
The frequency-dependent storage modulus of MRP and Gr–MRP for different magnetic fields of 0 and 540 mT.

In this study, a frequency sweep test was performed in the range of 0.1 to 100 Hz under the oscillatory shear mode with magnetic field intensities in the off- and on-state conditions of 0 and 540 mT, under a fixed shear strain of 0.03%. The values of the storage modulus G’ of MRP and Gr–MRP were obtained from this measurement and are shown in Figure 10. As shown in this figure, in the off-state condition, the MPR exhibits the lowest storage modulus and this increased dramatically in the on-state condition, For the Gr–MRP samples, the storage modulus slightly increased with an increase in the magnetic field along with an increase in frequency. In addition, it can clearly be seen that the Gr–MRP samples have a larger initial storage modulus and a higher storage modulus across the range than the MRP samples. This might be due to the fact that the addition of Gr increased the rigidity of the MRP, thus contributing to the increased storage modulus at all applied frequencies [43]. In addition, the increase in the storage modulus with an increasing magnetic field was attributed to the strengthening of the chain alignment of particles (CIPs and Gr), leading to the MRP becoming stiffer.

## 4. Conclusions

In this study, seven samples, including PVA, MRP, and MRP containing different amounts of Gr (6 to 10 wt.%) were fabricated, and their resistivity properties were successfully investigated. The material characterizations were undertaken by investigating the relationships between the resistivity, conductivity, and magnetic flux density. From the results, an improved conductivity effect of Gr–MRP was observed at the highest Gr content of 10 wt.%. Thus, it was identified that a stronger interfacial interaction between the additive and the matrix can contribute to a lower resistivity. The electrical conductivity characteristic was enhanced by adding Gr to the microstructures, simultaneously strengthening the CIP chain structure. Therefore, the effect of resistivity in the Gr–MRP samples investigated in this work means that they can potentially be used for a wide variety of applications as soft and flexible sensors. The main results achieved in this work are summarized as follows.

(1) The Gr–MRPs were fabricated using 6 wt.% to 10 wt.% Gr containing a DMSO/water ratio of 80:20 by weight. The magnetic properties of all samples were experimentally investigated. The results showed that the Gr–MRP sample had a higher M_s_ of 61.15 emu/g compared to the MRP sample (42.45 emu/g). The M_s_ of Gr–MRP dropped by 45% in comparison to the MRP sample, due to the Gr additive, which may hinder and obstruct the movement of CIPs. Thus, the interparticle interactions resulted in a smaller value of M_s_.
(2) The resistivities of the Gr–MRP samples decreased with increasing magnetic flux density. The higher the Gr content, the lower resistivity value observed. The conductivity of 10 wt.% Gr–MRP was found to be the highest and was 178.06% higher than the Gr–MRP with 6 wt.% at a magnetic flux density of 400 mT. In MRP, Gr functioned as the conductive agent and contributed to the improvement of the continuous conducting network.
(3) The effective interfacial interaction between the additive and matrix increased the storage modulus of Gr–MRP, with a stronger interaction resulting in a higher storage modulus for Gr–MRP than for MRP only.

The results presented in this work are self-explanatory, showing that Gr additives can improve the electrical conductivity, which is a crucial factor in the use of these materials as a potential sensor. However, the relationships between the field-dependent properties such as complex moduli and the reinforcing mechanism of Gr in PVA-based MRP need to be further explored, in order to develop flexible sensors for diverse practical applications. In addition, an FT-IR experiment will be undertaken to investigate the electrical and magnetic behaviors associated with the mechanism of the movement of the Gr and CIPs. 

## Figures and Tables

**Table 1:** The composition of each hydrogel MRP sample.

Type	Samples	CIP (wt.%)	PVA Matrix (wt.%)	CIP (wt.%)
MRP	1	70	30	0
Gr–MRP	2	70	24	6
-	3	70	23	7
-	4	70	22	8
-	5	70	21	9
-	6	70	20	10

**Table 2:** Magnetic properties of MRP and Gr–MRP samples.

Samples	H_c_ (kA/m)	M_s_ (emu/g)	M_r_ (emu/g)
MRP	7.97	61.15	200.67 × 10^−2^
Gr–MRP	7.97	42.45	116.58 × 10^−2^

**Table 3:** The resistivity values of PVA and MRP under different magnetic flux densities.

	Samples	Magnetic Flux Density, mT				
		0	100	200	300	400
Resistivity (σ), kΩ·m	PVA	1.15 × 10^6^	1.15 × 10^6^	1.15 × 10^6^	1.15 × 10^6^	1.15 × 10^6^
	MRP	1.05 × 10^6^	1.04 × 10^6^	1.00 × 10^6^	9.90 × 10^6^	9.90 × 10^6^

**Table 4:** The resistivity values for various weight percentages of Gr.

	Samples	Magnetic Flux Density, mT				
		0	100	200	300	400
Resistivity (σ), kΩ·m	2	9.90 × 10^4^	8.16 × 10^4^	5.97 × 10^4^	4.46 × 10^4^	3.74 × 10^4^
	3	6.87 × 10^4^	4.25 × 10^4^	3.19 × 10^4^	2.35 × 10^4^	1.90 × 10^4^
	4	5.05 × 10^4^	3.25 × 10^4^	1.91 × 10^4^	1.80 × 10^4^	1.76 × 10^4^
	5	4.16 × 10^4^	2.84 × 10^4^	1.37 × 10^4^	1.36 × 10^4^	1.35 × 10^4^
	6	4.14 × 10^4^	2.43 × 10^4^	1.36 × 10^4^	1.36 × 10^4^	1.35 × 10^4^

## Data Availability

The raw/processed data required to reproduce these findings cannot be shared at this time as the data are also part of an ongoing study.
